# Refractory and/or Resistant Herpes Simplex Virus Mucocutaneous Infections in Adult Transplantation and Cellular Therapy Patients: A Single-Center Experience

**DOI:** 10.7759/cureus.110131

**Published:** 2026-06-02

**Authors:** Joseph I Berger, Anthony D Anderson, Octavio V Martinez, Michele I Morris, Mohammed Raja, Lazaros J Lekakis, Amer Beitinjaneh, Antonio Jimenez, Mark Goodman, Trent P Wang, Jay Spiegel, Noa G Holtzman, Krishna V Komanduri, Denise Pereira, Damian Green, Jose F Camargo

**Affiliations:** 1 Division of Infectious Diseases, University of Miami, Miami, USA; 2 Division of Pharmacology, Sylvester Comprehensive Cancer Center, Miami, USA; 3 Division of Pathology and Laboratory Medicine, University of Miami, Miami, USA; 4 Division of Transplantation and Cellular Therapy, Sylvester Comprehensive Cancer Center, Miami, USA; 5 Division of Hematology and Oncology, University of California San Francisco Medical Center, San Francisco, USA

**Keywords:** car-t, hematopoietic cell transplantation, herpes simplex virus, refractory, resistant

## Abstract

Purpose and methods

Refractory and/or resistant herpes simplex virus (R/R HSV) mucocutaneous infections are an emerging challenge in immunocompromised hosts. A single-center retrospective study of R/R HSV mucocutaneous infections in allogeneic hematopoietic cell transplantation (HCT) was conducted on cases identified between 2023 and 2025. Cases were categorized using the 2025 consensus definitions. We collected data on the clinical presentation, risk factors, and outcomes and investigated the genetic mechanisms of resistance.

Results

Five cases of R/R HSV following HCT were identified. Four patients had documented *UL23 *gene resistance mutations. The median time from HCT to R/R HSV was 642 days. All the patients were recipients of unrelated or mismatched donors. Three (60%) had relapsed hematological disease. The CD4 lymphopenia (<200 cells/µL) and hypogammaglobulinemia (IgG <600 mg/dL) were present in three patients (60%) and four patients (80%), respectively. The median cumulative exposure to (val)acyclovir prior to R/R HSV was 30 months (range: 18 to 51 months). Foscarnet was used in all patients as initial therapy. Viremia was present in four (80%) cases, with clearance of viremia in response to antiviral therapy in only two. Acute renal failure and electrolyte disturbances related to antiviral therapy occurred in two patients (40%) and four patients (80%), respectively. Despite protracted courses of antivirals (lasting up to nine weeks), no patient achieved complete healing. Three-month mortality was 80% (n=4), although none of the deaths were directly attributed to the HSV infection.

Conclusion

Refractory and/or resistant herpes simplex virus is a rare but serious complication of HCT. Current antivirals for R/R HSV infections have suboptimal efficacy and safety profiles.

## Introduction

Herpes simplex virus (HSV) infections are highly prevalent in the immunocompromised host. The seroprevalence among the adult general population is estimated to be 50% to 65% for HSV‐1 and 10% to 20% for HSV‐2 [1]. These infections account for nearly 50% of oropharyngeal lesions in the early post-transplant phase; however, systematic antiviral prophylaxis in these patients has resulted in a dramatic reduction in the incidence of HSV infections, decreasing from 80% to 10% in hematopoietic cell transplantation (HCT) patients [2]. 

Long-term acyclovir is well tolerated, but the drawback of universal prophylaxis is that prolonged or repeated exposure to antiviral agents can lead to the emergence of resistant strains [3]. Mutations in the* UL23* gene encoding for the thymidine kinase (TK) account for 95% of known resistance mutations to acyclovir; the *UL30* gene (DNA polymerase, which is the common target for all nucleoside analogues, as well as foscarnet and cidofovir) mutations account for only 5% of known resistance mutations to acyclovir but can confer resistance to other classes of antivirals [2]. The prevalence of drug resistance is significantly higher among HSV-2 isolates [4]. While resistance mutations usually result in a reduction in viral fitness and virulence, infections remain sufficient to cause severe forms in profoundly immunocompromised individuals [2].

The prevalence of acyclovir-resistant HSV in allogeneic HCT can be as high as 17% to 28% [5,6], occurring mostly in the setting of T cell depletion or graft-vs-host disease (GVHD) [7,8]. Hematological malignancy (particularly relapsed disease), HIV infection, hypogammaglobulinemia, and corticosteroid use are also common risk factors [9,10]. Presentations may range from localized (typically oral or anogenital) to disseminated disease, allowing for significant morbidity and mortality, and atypical presentations can occur [9,11].

Medical management of patients with refractory and/or resistant (R/R) HSV infections can be particularly challenging given the limited treatment options available, often requiring long courses of antiviral therapy (up to 60-80 days in various cases) and prolonged hospitalization [5,9,12]. The response to the standard-of-care antiviral in this setting, foscarnet, is only partial at best (<50%) with therapy-associated electrolyte disturbances (65%) and acute kidney dysfunction (42% to 86%) limiting its use [5,9,12]. This highlights the need for novel antivirals. Recently, consensus definitions of R/R HSV mucocutaneous infections have been proposed with the hope of facilitating clinical trials in immunocompromised hosts [8]. Here, we aimed to apply the new 2025 consensus definitions to a real-world transplant population and describe the clinical characteristics and outcomes of allogeneic HCT patients with R/R HSV using the new proposed classificatory criteria.

## Materials and methods

This is a single-center retrospective study of consecutive patients with R/R HSV following HCT identified between 2023 and 2025. This study aimed to evaluate the clinical presentation, resistance mechanisms, treatment outcomes, and associated complications of R/R HSV infections in allogeneic HCT recipients at a single center. Consecutive patients were included if they met the 2025 R/R HSV classificatory criteria [8]. Per our institutional protocol, all HCT patients receive universal acyclovir prophylaxis at 800 mg PO twice a day for a minimum of 12 months, until they are off immunosuppression and have completed their recombinant zoster vaccine series [13]. This study was reviewed by our Institutional Review Board, the University of Miami Human Subject Research Office, and deemed to be exempt. The study was conducted in accordance with the principles set forth in the Declaration of Helsinki.

Genotypic acyclovir resistance testing was conducted in clinical specimens (i.e., mucocutaneous lesion swabs) at the University of Washington Department of Laboratory Medicine and Pathology using Sanger sequencing to detect specific mutations found in the *UL23* gene encoding for the TK of both HSV type 1 and HSV type 2. Sequence analysis was performed on the full-length *UL23* gene, amino acids 1 to 376, for HSV. The assay sensitivity is 1,000 copies/mL.

Cases were categorized as R/R HSV using the 2025 consensus classificatory criteria [8]. Cases were reviewed and adjudicated by two infectious diseases specialists (MR and JFC) before being included in the study for analysis. Refractory disease was defined as HSV-positive mucocutaneous lesion(s) with failure to improve clinically after at least seven days of appropriate therapy or new HSV-positive mucocutaneous lesion(s) after receiving appropriately dosed therapy for at least seven days. Resistant infections were defined as refractory HSV infections with viral genetic alteration(s) that decreased susceptibility and/or phenotypic assays demonstrating an increased EC50 above the assay cutoffs to one or more antiviral drugs, in the absence of other causes of mucositis [8]. Chart review was conducted for each study subject to retrospectively collect clinical data, including demographics, details about immunosuppression and underlying risk factors, duration of antiviral prophylaxis, resistance mutations, antiviral and adjuvant therapies, response to therapy, and mortality.

## Results

**Figure 1:**
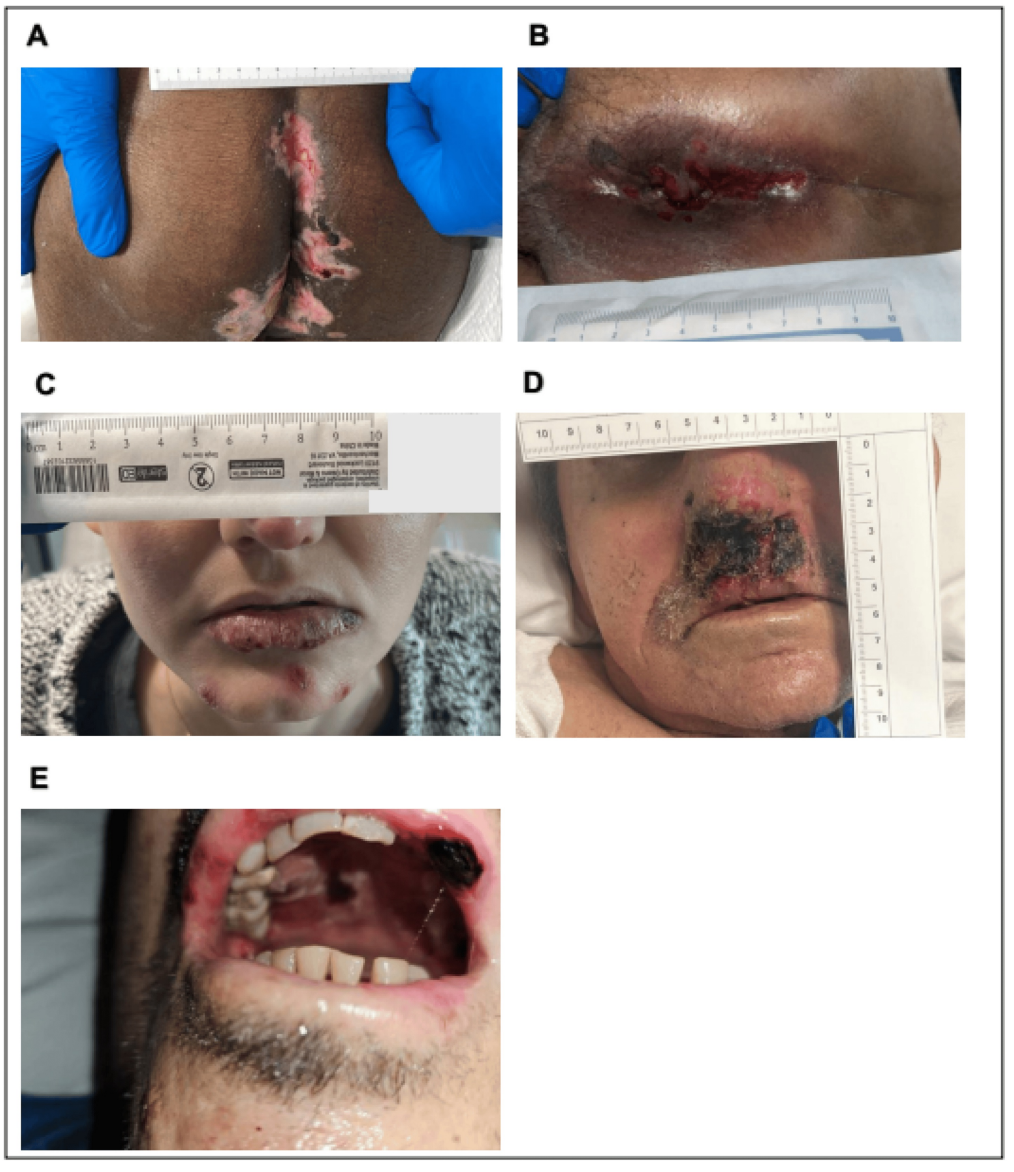
R/R HSV mucocutaneous infections in transplantation and cellular therapy (TCT) patients A: Perirectal HSV in patient #1; B: Perirectal HSV in patient #2; C: Orolabial HSV in patient #3; D: Nasolabial HSV in patient #4; E: Orogingival HSV in patient #5 R/R HSV: Refractory and/or resistant herpes simplex virus;  TCT: Transplantation and cellular therapy

We identified five cases of R/R HSV in allogeneic HCT patients. Two of these patients also received chimeric antigen receptor (CAR)-T cell therapy. The clinical characteristics, laboratory data, and outcomes of these five cases are shown in Table 1. The median time from transplant to R/R HSV was 642 days (range: 95-981 days). Four (80%) of the cases were male, and three (60%) were White Hispanic. The median age was 55 years (range: 37-74 years). Three cases corresponded to HSV-1, all with oral or facial involvement, and two cases to HSV-2, both perirectal (Figure 1). Four patients had documented *UL23 *gene resistance mutations (specific codon mutations shown in Table 1). The median turnaround time from sample collection to resistance genotypic results was 15 days (range: 11-19 days). All the patients were HCT recipients of unrelated or human leukocyte antigen (HLA)-mismatched donors. 

**Table 1:** Characteristics of TCT patients with R/R HSV mucocutaneous infections ^a^Clinically significant CMV viremia in the three months prior to R/R lesion; ^b^Systemic immunosuppression/chemotherapy at the time of presentation or within three months prior; ^c^Drug-associated AKI defined as greater than ≥0.3 mg/dL in 48 hrs or 1.5x baseline during either foscarnet or systemic cidofovir treatment for confirmed resistant or refractory HSV; ^d^Drug-associated electrolyte disturbances defined as any potassium, magnesium or phosphorus level below the reference range which required repletion to correct during either foscarnet or systemic cidofovir treatment for confirmed resistant or refractory HSV; ^e^None of the deaths was directly attributable to HSV infection TCT: Transplantation and cellular therapy; R/R HSV: Refractory and/or resistant herpes simplex virus; GVHD: Graft-vs-host disease; ATG: Antithymocyte globulin; CMV: Cytomegalovirus; HSV: Herpes simplex virus; AML: Acute myeloid leukemia; AKI: Acute kidney injury; B-ALL: B-cell acute lymphoblastic leukemia; CAR-T: Chimeric antigen receptor T-cell therapy; MMUD: Mismatched unrelated donor; MUD: Matched unrelated donor; R/R: Refractory/resistant; T-ALL: T-cell acute lymphoblastic leukemia; TCL: T-cell lymphoma

Characteristics	Patient #1	Patient #2	Patient #3	Patient #4	Patient #5
Age/gender	55M	54M	37F	74M	72M
Race/ethnicity	Black	White (Hispanic)	White (Hispanic)	White (Hispanic)	White
Underlying disease	Hepatosplenic T-cell lymphoma (TCL)	Acute myeloid leukemia (AML)	B-cell acute lymphoblastic leukemia (B-ALL)	AML	T-cell acute lymphoblastic leukemia (T-ALL)
HIV-1 infection	No	Yes (viral load suppressed)	No	No	No
GVHD 2-4	No	No	No	No	Yes
Type of TCT	Allogeneic mismatched unrelated donor (MMUD)	Allogeneic MMUD	Allogeneic matched unrelated donor (MUD) > CD19 chimeric antigen receptor T-cell therapy (CAR-T)	Allogeneic MUD	CD4 CAR-T > allogeneic MMUD
Antithymocyte globulin (ATG) during conditioning	No	No	Yes	Yes	No
Malignancy status	Relapse	Remission	Relapse	Relapse	Remission
CMV (D)onor/(R)ecipient serostatus	D+/R-	D-/R+	D-/R+	D-/R+	D-/R-
Clinically significant CMV^a^	No	No	No	No	No
Systemic immunosuppression/chemotherapy^b^	Duvelisib romidepsin	None	Venetoclax cyclophosphamide vincristine	Venetoclax decitabine	Prednisolone sirolimus
CD4 count <200 cells/µL	Yes	Yes	No	N/A	Yes
CD19 count <50 cells/µL	No	Yes	N/A	N/A	Yes
CD56 count <50 cells/µL	No	No	N/A	N/A	Yes
IgG levels <600 mg/dL	Yes	Yes	Yes	N/A	Yes
HSV prophylaxis	Valacyclovir	Valacyclovir	Acyclovir	Acyclovir	Acyclovir
R/R HSV location	Perirectal	Perirectal	Orolabial	Nasolabial	Orogingival
Time from HCT to R/R HSV, days	642	722	567	981	95
Resistant HSV type	HSV-2	HSV-2	HSV-1	HSV-1	HSV-1
R/R HSV	Yes/yes	Yes/yes	Yes/no	Yes/yes	Yes/yes
Resistant *UL23 *mutations	Glu147Gly	Ala310Cys	None	Glu146Gly	A93V
Other *UL23* mutations causing amino acid changes	R26H, G39E, N78D	G39E	A17V, A124P	None	C6G, L42P, Q89R, V348I
HSV DNAemia duration, days	7	N/A	8	>90	>15
Acyclovir exposure prior to presentation, months	24.3	30.4	18.2	37.8	50.9
R/R HSV Treatment	Foscarnet cidofovir	Foscarnet cidofovir	Foscarnet	Foscarnet	Foscarnet cidofovir
Duration of systemic antiviral therapy, weeks	9	5	2	9	5
Topical treatment	Cidofovir 1%	Cidofovir 1%	None	Imiquimod 5%	None
Foscarnet/IV cidofovir-associated acute kidney injury (AKI)^c^	Yes	No	No	No	Yes
Foscarnet/IV cidofovir-associated electrolyte disturbances^d^	Yes	Yes	No	Yes	Yes
IVIG	Yes	Yes	Yes	Yes	No
Response to foscarnet/cidofovir	Partial	Partial	Partial	Partial	Partial
Cause of death	Hospice	Post-procedural	Acute hypoxic respiratory failure	Hospice	Septic shock
Three-month survival	No	No	No	No	Alive
12-month survival^e^	No	No	No	No	No

**Figure 2:**
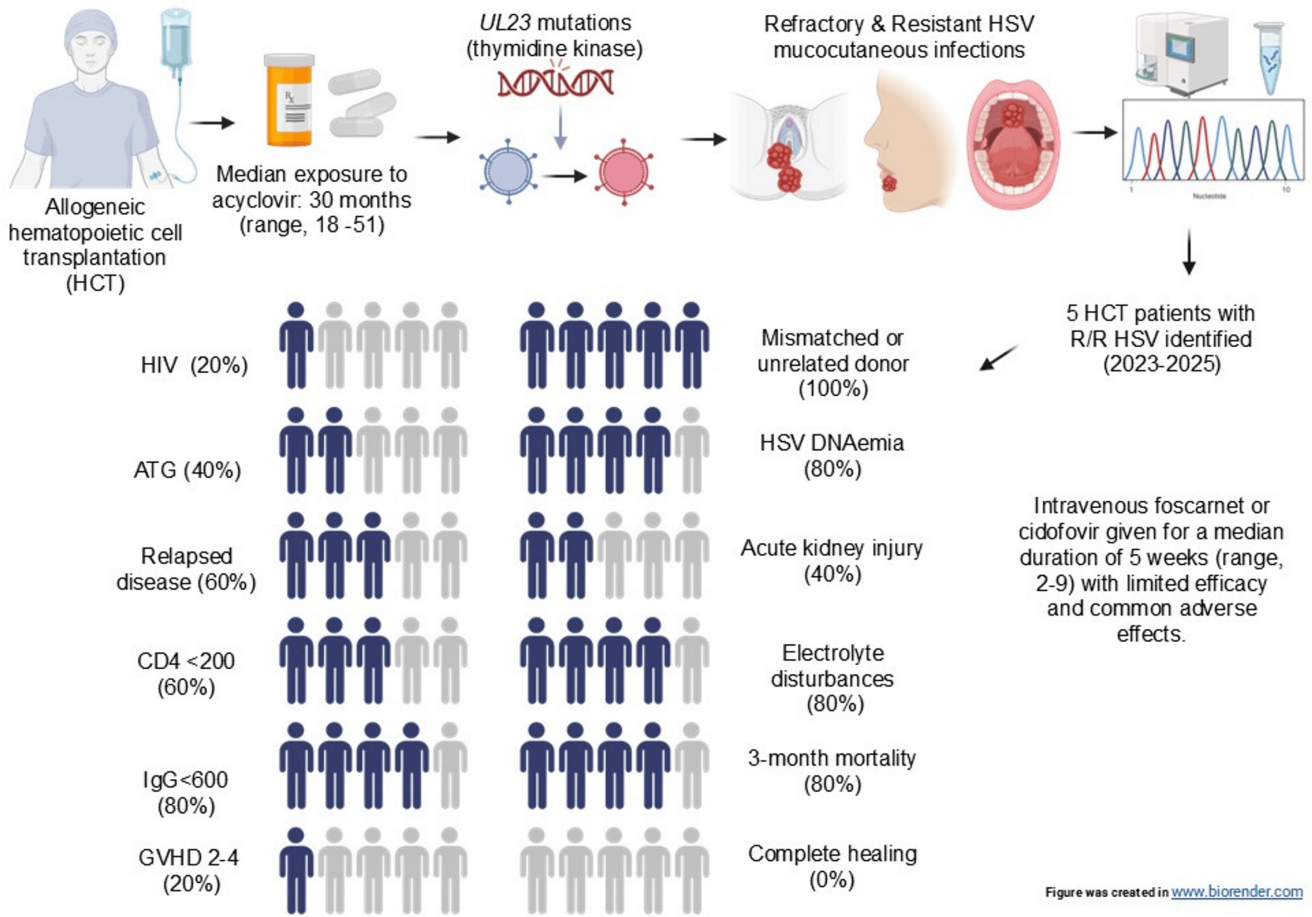
Visual abstract This illustration was created by the authors using BioRender core tools sans generative AI. R/R HSV: Refractory and/or resistant herpes simplex virus

Underlying HIV-1 or GVHD grade 2-4, requiring systemic immunosuppressive therapy, was present in one patient each. Three (60%) patients had relapsed hematological disease and received chemotherapy within 90 days prior to the R/R HSV diagnosis. The CD4 lymphopenia (< 200 cells/µL) and hypogammaglobulinemia (IgG < 600 mg/dL) were present in 60% and 80% of the cases, respectively (Figure 2). All patients were on prophylactic acyclovir (or its prodrug, valacyclovir) prior to R/R HSV diagnosis. The median cumulative exposure to (val)acyclovir prior to presentation with R/R HSV was 30 months (range: 18-51 months).

Foscarnet was used in all patients as initial therapy, whereas intravenous cidofovir was used as step-down therapy in two patients. Four of five patients received adjunctive intravenous immunoglobulins (IVIG). Viremia (HSV DNAemia) was present in four (80%) cases, with clearance of viremia in response to antiviral therapy in only two. The median time to resolution of viremia in these two cases was 7.5 days (range: seven to eight days). Two patients (40%) developed acute renal failure related to antiviral therapy requiring dose adjustments for creatinine clearance, and electrolyte disturbances were common (80%) (Figure 2). Protracted courses of antivirals (foscarnet followed by cidofovir, or foscarnet alone) were required, with a median duration of therapy of five weeks (range: two to nine weeks); and although clinical improvement was noted in all the patients, complete healing and resolution of the infection were achieved in none. Three-month mortality was 80%, and none of the patients survived 12 months from the R/R HSV diagnosis, although none of the deaths were directly attributed to the HSV infection. Causes of death included acute respiratory failure, procedure-related mortality, septic shock, and elective hospice with withdrawal of care. 

## Discussion

Although rare in the immunocompetent (0.1%-0.7%), acyclovir-resistant HSV has been reported in 3.5%-7% of HIV-positive patients and 2%-3% of solid organ transplant recipients (as high as 10% in some studies) [2]. The highest prevalence has been reported in HCT with rates of 4.1% to 10.9% (but as high as 28%-36% in some studies) [6,14,15]. In a report of over 20 studies, the median prevalence of acyclovir-resistant HSV infections was 16.1%, and it appeared to be significantly increasing in recent years [2]. It must be noted that the five cases presented here were identified within a three-year window, further supporting the increase in incidence of R/R HSV in recent years. The increased incidence of R/R HSV could be due to improved awareness among clinicians of acyclovir resistance; higher availability and use of genotypic testing; widespread implementation of universal antiviral prophylaxis in transplant programs; and the overall growth of the immunocompromised population [16].

The HCT recipients are heavily exposed to acyclovir for the prevention of HSV and varicella zoster virus (VZV) during chemotherapy in the pre-transplant period and, subsequently, for a minimum of six to 12 months post-HCT [13], sometimes longer in the setting of maintenance chemotherapy, relapsed hematological disease, or chronic GVHD. In addition to prolonged antiviral exposure with a median of 30 months of (val)acyclovir prior to presentation, the five patients described here had many of the known risk factors for R/R HSV in HCT, including unrelated or HLA-mismatched donors, the use of T-cell depletion (i.e., antithymocyte globulin (ATG)), myeloid malignancies, relapsed disease, and the presence of GVHD requiring corticosteroids [2,6,7].

Acyclovir-refractory HSV infection is a clinical diagnosis that requires attention to host type (e.g., allogeneic HCT, relapsed disease, GVHD); prolonged acyclovir exposure (typically > 24 months); and lack of response to treatment dose valacyclovir or IV acyclovir after seven days of therapy [8]. A definitive diagnosis of acyclovir-resistant HSV can be made via phenotypic testing (i.e., plaque reduction assay), which is limited by prolonged turnaround time (in the range of several weeks), as this method requires isolation of viral strains in cell culture; and genotypic testing for *UL23* mutations [2], which, although not widely available in the U.S., can be performed at the University of Washington molecular laboratory with a turnaround time, in our experience, of about two weeks. Rapid diagnostic tests are therefore needed in this area. As a comparison, the current turnaround time for genotypic cytomegalovirus (CMV) resistance testing at our reference commercial laboratory is around four to five business days. Given the current turnaround times for genotypic HSV resistance testing, and considering the severity and high morbidity/mortality associated with R/R HSV, antiviral therapy should not be delayed while confirmatory genotypic testing is obtained.

Management of acyclovir-resistant HSV is challenging for various reasons, including host factors, as R/R HSV is more common in highly vulnerable immunocompromised individuals (typically after multiple lines of chemotherapy and HCT) with comorbidities (e.g., HIV, hypogammaglobulinemia); few available antiviral agents with limited efficacy for the treatment of R/R HSV. Despite prolonged courses of foscarnet or cidofovir, with long hospital stays, only half of the patients presented here had clearance of HSV viremia, and none achieved complete healing of the mucocutaneous lesions. While clinical response was only partial, drug toxicity, primarily nephrotoxicity and electrolyte disturbances, was common. 

The first cases of HSV resistance to acyclovir were reported in the literature in 1982 [2]. More than four decades later, there is still a paucity of antiviral agents with activity against TK-deficient (*UL23* mutant) HSV strains. Brincidofovir is an orally available lipid conjugate of cidofovir with broad antiviral activity against double-stranded DNA viruses that, unlike cidofovir, is not nephrotoxic [17,18]. Brincidofovir has been successfully used for the treatment of R/R HSV [19], but this agent is only approved in the U.S. for smallpox and must be obtained through FDA emergency use authorization. 

The helicase-primase complex is responsible for unwinding the DNA strands and priming the replication process [8]. Pritelivir, a helicase-primase inhibitor (HPI), is a novel antiviral against both HSV-1 and HSV-2. Since pritelivir does not require activation by viral thymidine kinase, it retains antiviral activity against *UL23* mutant acyclovir-resistant HSV strains. Indeed, successful treatment of R/R HSV with compassionate use pritelivir has been reported in six allogeneic HCT recipients [20-23]. In a phase 2 clinical trial, pritelivir had a higher rate of lesion healing compared to foscarnet in immunocompromised patients with R/R HSV [24]. In the PRIOH-1 phase 3 trial, pritelivir demonstrated superior lesion healing of 63% vs. 34% compared to the investigator's choice therapy [25]. Of note, pritelivir lacks antiviral activity against other herpes viruses such as VZV [2], and therefore it should be co-administered with acyclovir. The combination of pritelivir with acyclovir suppresses the evolution of HSV resistance *in vitro* [26]. Although pritelivir is not yet FDA-approved, it is currently undergoing priority review for the treatment of HSV infections in immunocompromised patients. In a recent report from the UK, involving eight cases of R/R HSV in pediatric allogeneic HCT recipients, two of their patients received compassionate use of pritelivir [10]. The authors comment on how there was a significant delay (> 20 days) between application, approval, and arrival of the drug. Likewise, we attempted to obtain pritelivir for patient #4, but his enrollment into the U.S. Expanded Access Program/clinical trial was precluded due to strict exclusion criteria (including cytopenias) and the fact that our patient was not in optimal condition to be discharged from the hospital for the required outpatient evaluation. Amenamevir is another HPI that has been successfully used for R/R HSV in allogeneic HCT patients, but it is only approved in Japan [27,28]. Thus, timely access to HPI for treatment of R/R HSV remains an unmet need in the highly vulnerable HCT patient population. 

Although hypogammaglobulinemia was present in a quarter of the patients in recent studies [9] and up to 80% of the cases presented here, it is unclear whether administration of IVIG has any role in the treatment of R/R HSV. Since tissue injury in HSV disease is driven by immune reactions stimulated by HSV rather than HSV itself, a potential role of immunomodulatory therapy with IVIG has been proposed recently [29]. Adoptive T-cell immunotherapy holds great promise for the treatment of viral complications in immunocompromised patients resistant to standard antiviral strategies [30-33]. Successful use of HSV-1-specific T cells from a partially HLA-matched donor for severe acyclovir-resistant HSV-1 mucositis has been reported, but experience with adoptive T-cell therapy for HSV remains anecdotal at best [34].

Further considerations for R/R HSV include topical therapies. Specifically, topical imiquimod has the ability to stimulate the production of IFN-γ, TNF-α, and IL-12 [35]. Additionally, compounded versions of topical cidofovir have been used with some success [36,37]. In our experience, topical imiquimod was not well tolerated, as the patient experienced local irritation with discomfort after application, and topical cidofovir use was limited by logistic difficulties, as it requires a compounding specialty pharmacy, and it is not cost-effective for patients with large (i.e., more than a few centimeters) mucocutaneous lesions.

Currently, no guidance exists on whether secondary prophylaxis for acyclovir-resistant HSV is indicated in these scenarios, and this merits further study. An ideal prophylactic agent should be affordable, well-tolerated, effective, and have a relatively high threshold for the emergence of resistance. 

Although current consensus guidelines for clinical trials define refractory HSV infection as failure to improve clinically after at least seven days of appropriately dosed, directed anti-HSV therapy [8], considering i) the severity of the cases presented here and the intense and incapacitating discomfort that is characteristic of R/R HSV lesions; and ii) the fact that early antiviral therapy is a fundamental principle in the management of viral infections in general (including influenza, SARS-CoV2, CMV, and HIV), we concur with others [9,10] that in a 'real-world' scenario, an alternative antiviral should be considered in patients who do not show signs of improvement after three to five days of anti-HSV therapy. 

The strengths of this study lie in applying the newest consensus guidelines to better define R/R HSV cases in our patient population. Although traditionally a clinical diagnosis, the increased access to genotypic testing also facilitated confirmation of *UL23* mutations in most of these cases. We provide contemporary data on the clinical presentation and outcomes of this condition. This study is limited due to its single-center nature, small number of patients, and potential risk of selection bias. However, our results set the foundation for future prospective and/or multicenter studies in this area.

## Conclusions

In conclusion, R/R HSV is an emerging challenge in allogeneic transplant recipients. Diagnosis requires a high index of clinical suspicion based on established risk factors and prolonged antiviral exposure. Current antivirals for R/R HSV infections have a suboptimal efficacy and safety profile. Broader availability of genotypic testing and improved access to novel, more effective, and better-tolerated antivirals represent current unmet needs in this highly vulnerable patient population.
